# Reactive mesothelial hyperplasia mimicking mesothelioma in an African green monkey (*Chlorocebus aethiops*)

**DOI:** 10.5194/pb-7-5-2020

**Published:** 2020-06-15

**Authors:** Roland Plesker, Kernt Köhler, Susanne von Gerlach, Klaus Boller, Markus Vogt, Inke S. Feder

**Affiliations:** 1 Paul-Ehrlich-Institut, Paul-Ehrlich-Str. 51–59, 63225 Langen, Germany; 2 Institut für Veterinär-Pathologie, Justus-Liebig-Universität Gießen, Gießen, Germany; 3 ÜGP MVZ, Institut für Pathologie, Zytologie und Molekularpathologie GbR, Wettenberg, Germany; 4 Institut für Pathologie, Ruhr-Universität Bochum, Bochum, Germany

## Abstract

A spontaneous reactive mesothelial hyperplasia occurred in a female, 15.7-year-old African green monkey (grivet; *Chlorocebus aethiops*). At necropsy, massive effusions were found in the abdomen, the thorax, and the pericardium. Additionally, multiple small, beige-gray nodules were detected on the serosal surfaces of the abdominal organs. Histopathologically, the mesothelial cells resembled the epithelioid subtype of a mesothelioma, but no infiltrative or invasive growth could be demonstrated. The mesothelial cells on the thoracis, liver, and intestinal serosa were accompanied by chronic serositis. Mesothelial cells expressed cytokeratin, vimentin, calretinin, desmin, Wilms Tumor 1 (WT-1) protein, and epithelial membrane antigen (EMA). Cells were negative for carcinoembryonic antigen (CEA), cluster of differentiation 15 (CD15), and podoplanin. Ultrastructurally, cells revealed a moderate amount of microvilli of medium length, perinuclear tonofilament bundles, and long desmosomes. In fluorescence in situ hybridization (FISH) for the detection of characteristic gene loss (p16; CDKN2A), NF2, and MTAP, no deletions were detected. No asbestos fibers and no presence of Simian virus 40 antigen (SV40) could be demonstrated.

## 1 Introduction and literature overview

### 1.1 Mesothelial hyperplasia

Mesothelial hyperplasia is a benign, reactive condition with no neoplastic potential that is associated with a variety of chronic and acute injuries to the mesothelial surface (Watkins et al., 2018). It can occur as a response to inflammatory, infectious, toxic, or neoplastic triggers (Losada et al., 2018). For example, mesothelial cells might undergo hyperplasia as a response to serositis (Barth et al., 1997). Macroscopically, it may manifest as small white nodules (Watkins et al., 2018).

Besides humans, mesothelial hyperplasia is reported in monkeys (Sato et al., 2012; Winn et al., 2018), dogs (Milne et al., 2018), cats (Weiss and Scott, 1981), rabbits (Dalton and Chun, 1966), horses (Hoon-Hanks et al., 2016), cattle (Goldsmith and Adaska, 2020), rats (Slater et al., 1991), and mice (Adissu et al., 2015).

Histologically, reactive mesothelial proliferations may mimic mesothelioma because they may show high cellularity, numerous mitotic figures, cytologic atypia, necrosis, papillary growth, and entrapment of mesothelial cells within fibrosis mimicking invasion (Husain et al., 2018).

### 1.2 Mesothelioma

Mesotheliomas on the other hand are primary tumors of the serosa (Hammar, 1994) and uncommon in animals and humans (Fortman et al., 1993). The occurrence of mesotheliomas in man often had been associated with asbestos (Feder et al., 2018) and – to a lesser extent – other mineral fibers, Simian virus 40 infection, or radiation (Attanoos et al., 2018). Malignant mesotheliomas have been reported in dogs (D'Angelo et al., 2014), cats (Heerkens et al., 2011), cattle (Takkasu et al., 2006), goats and horses (Head et al., 2002), mice (Robinson et al., 2014), rats (Davis, 1979), and hamsters (Kroczynska et al., 2006; Kane, 2006). In monkeys, mesotheliomas have been induced experimentally by inhalation of amosite asbestos or by diethylstilbestrol administration (Webster et al., 1993; McClure and Graham, 1973). In 2007, Yamate et al. published a report on a spontaneous peritoneal malignant mesothelioma in a geriatric Japanese macaque (*Macaca fuscata*). A spontaneous pericardial mesothelioma was reported in a rhesus monkey (*Macaca mulatta*) by Chandra and Mansfield (1999). Fortman et al. (1993) described a malignant mesothelioma with fluids in the thoracic and the abdominal cavity of an olive baboon (*Papio anubis*).

### 1.3 Discrimination between benign and malignant mesothelial alterations

For discrimination between benign and malignant mesothelial alterations, neoplastic invasion or metastases are key features for a clear diagnosis of malignant mesothelioma (Husain et al., 2018). No tissue or serum marker (including the molecular detection of p16/CDKN2A) has been proven to have sufficient specificity, consistency, and reproducibility that it can replace evidence of invasion as the decisive marker for diagnosis (Henderson et al., 2013).

Nevertheless, helpful immunohistochemical markers that may aid in differentiating benign from malignant mesothelial alterations are p53, desmin, epithelial membrane antigen, glucose transporter 1, and U3 small nucleolar ribonucleoprotein protein (IMP-3) (Churg et al., 2016). However, although certain immunohistochemical stains are more likely to be positive in benign proliferations and others in malignant proliferations, these cannot be solely relied upon in the diagnosis of individual cases (Husain et al., 2018). Thus, definite diagnosis might therefore not be possible solely using histology and immunohistochemistry (Tischoff and Tannapfel, 2017).

However, the finding of homozygous deletion of p16 by fluorescence in situ hybridization (FISH) or the loss of BRCA1 associated protein 1 (BAP1) by immunohistochemistry is found only in mesotheliomas (Husain et al., 2018). Using gene-expression-based tests for BAP1 and p16 analysis might become a reliable tool to distinguish benign from malignant mesothelial proliferations (Ali et al., 2020).

Since sometimes it can be extremely difficult to distinguish between benign reactive mesothelial proliferation and mesothelioma, we here describe a spontaneous case of a mesothelial hyperplasia resembling a mesothelioma in an African green monkey (*Chlorocebus aethiops*). Immunohistochemistry, transmission electron microscopy, and genetic investigations were performed.

## 2 Animal and methods

### 2.1 Animal origin

The affected animal was a female, 15.7-year-old African green monkey (grivet; *Chlorocebus aethiops*). It was born at the Paul-Ehrlich-Institut in Langen, Germany, where it lived in an experimental indoor facility. It was group-housed in accordance with European and German animal welfare legislation. The monkey was used for experimental blood collection.

### 2.2 Animal housing

The cage was made of steel with a size of 300cm×375cm×225cm. Large windows allowed the monkey to watch the outside environment. Natural branches, ropes, nets, bedding, mirrors, kong toys, puzzle feeders, prima hedrons, music, and television were supplied for environmental enrichment. The diet consisted of monkey pellets ad libitum (Trio Munch^®^, Special Diet Services/Mazuri, Witham, England) in the morning and seasonal vegetables and fruits twice weekly in the afternoon. The monkey was also offered a mixture of nuts, mealworms, rice, popcorn, and curd.

### 2.3 Clinical history

There was no history of radiation in the medical records of this monkey. No unusual clinical observations were seen at the last visual veterinary control 3 d before the animal was found dead.

### 2.4 Necropsy and histology

Necropsy was performed immediately after the detection of the carcass. Photographs were taken and organs of interest were fixed in 4 % formaldehyde solution for 3 d before processing. Paraffin-embedding of fixed tissues, preparation of 4 µm sections, and hematoxylin–eosin (H&E)-staining were done in accordance with standard procedures. In addition, sections were also stained with periodic acid–Schiff (PAS) reaction. Prussian blue stain and Alcian blue stain were performed according to standard protocols (Riedelsheimer and Büchl-Zimmermann, 2015).

### 2.5 Further investigations

Parts of the lungs were ashed as described by de Vuyst et al. (1998) or Gibbs and Pooley (1996) in order to detect asbestos fibers.

### 2.6 Immunohistochemistry

In general, abdominal organs containing mesothelial proliferation on the surface were used for immunohistochemistry. African green monkey tissue of lung, pericardium, liver, and intestine and human tissue served as positive control.

The jejunum and the brain were tested for SV40.

Further details of immunohistochemistry are listed in Table 1.

**Table 1:** Antibodies for Immunohistochemistry.

Antibody directed against	Clone	Manufacturer	Dilution	Type of antigen retrieval	Detection system
Cytokeratin	Mouse monoclonal, panAB3 (Lu5), M9-744-A	Thermo Fisher	1:200	HIER*, Citrate (pH 6)	avidin-biotin-complex, DAB, Vector Lab. Burlingame, CA
Vimentin	Mouse monoclonal, V9, M0725	Dako Diagnostica, Hamburg	1:50	none	avidin-biotin-complex, DAB, Vector Lab. Burlingame, CA
Calretinin	Rabbit, polyclonal, RBK003	Zytomed Systems	1:300	HIER/Trilogy (pH 8.4)	Immonologic Bright Vision, medac GmbH, Wedel, Germany
Desmin	Mouse monoclonal, D33, 243M-16	Cell Marque	1:100	HIER/Trilogy (pH 8.4)	Immonologic Bright Vision, medac GmbH, Wedel, Germany
WT-1 protein (Wilms Tumor 1 protein)	Mouse monoclonal, 6F-H2, 348M-96	Cell Marque	1:100	HIER/Trilogy (pH 8.4)	Immonologic Bright Vision, medac GmbH, Wedel, Germany
EMA (epithelial membrane antigen)	Mouse monoclonal, E29, 247M-96	Cell Marque	1:100	HIER/Trilogy (pH 8.4)	Immonologic Bright Vision, medac GmbH, Wedel, Germany
Podoplanin	Mouse monoclonal, D2-40, 322M-16	Cell Marque	1:100	HIER/Trilogy (pH 8.4)	Immonologic Bright Vision, medac GmbH, Wedel, Germany
CD15 (cluster of differentiation 15) (CD 15-M)	Lab Vision CD15 Ab-3	Thermo Fisher	1:100	HIER, Citrate (pH 6)	Immonologic Bright Vision, medac GmbH, Wedel, Germany
CEA (carcinoembryonic antigen)	Mouse monoclonal, Col-1, MSK022	Zytomed Systems	1:50	Pronase	Immonologic Bright Vision, medac GmbH, Wedel, Germany
SV40 (Simian virus 40)	Mouse monoclonal IgG: T Ag (Pab 101) sc-147, Lot# C2613	Santa Cruz Biotechnology, Dallas, TX, USA	1:50	EDTA solution (pH 8.4)	streptavidin-biotin-complex DAB map kit, Roche Diagnostics, Germany

* HIER: heat induced epitope retrieval

### 2.7 Transmission electron microscopy

Formalin-fixed and paraffin-embedded tissue (as prepared for histology) was deparaffinized to 50 % alcohol, contrasted with 2 % uranyl acetate in 70 % alcohol, and plastic-embedded in EPON resin (Epoxy Embedding Kit, 45359-1EA-F; Sigma-Aldrich Chemie GmbH, Munich, Germany) according to standard procedures (Luft, 1961). Slides were examined using a JEM-1400 flash electron microscope (JEOL Ltd., Tokyo, Japan), and pictures were taken with a XAROSA camera (EMSIS, Münster, Germany) using the EMSIS RADIUS software.

### 2.8 Search for gene deletions

For the fluorescence in situ hybridization (FISH), formalin-fixed and paraffin-embedded material was sectioned at 2 µm, deparaffinized, and digested with pepsin for 15 min prior to another fixation. A FISH for the 9p21 locus (p16; CDKN2A) was performed using the ZytoLight^®^ SPEC CDKN2A/CEN 9 Dual Color Probe according to the manufacturer's instructions (ZytoVision GmbH, Bremerhaven, Germany). In the FISH for deletions in the neurofibromatosis type 2 gene (NF2) or the methylthioadenosine phosphorylase gene (MTAP), probes (NF2/CEN22q and MTAP/CEN9q) from Abnova (Taipei City, Taiwan) were used according to the supplier's instruction.

## 3 Results

### 3.1 Necropsy

**Figure 1:**
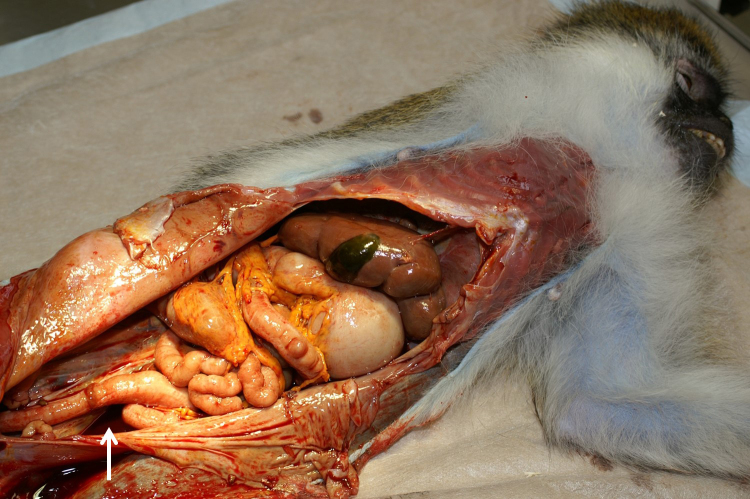
Opened abdominal cavity of an African green monkey with a reactive mesothelial hyperplasia: tan discoloration of the abdominal organs, rough appearance of the peritoneum, and remains of the abdominal fluid (arrow).

At necropsy, 700 mL of a dark-red to brown, turbid watery fluid was detected in the abdominal cavity. The thoracic cavity contained about 300 mL and the pericardial sac about 50 mL of fluid (Fig. 1). Additionally, within the fluid in the abdominal cavity, small beige flakes and a single free-floating gray to brown fibrin body measuring 7 mm in diameter were found.

**Figure 2:**
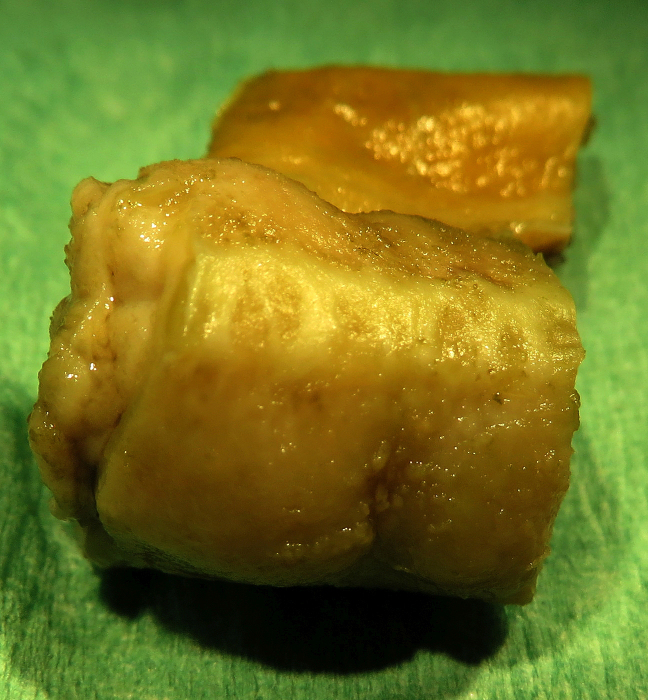
Formalin-fixed segment (1 cm) of the jejunum of an African green monkey with multiple small nodules on the serosal surface.

The peritoneal surface showed tan discoloration (Fig. 1). Multiple small, round beige-gray nodules (up to 0.5 mm) were detected on the serosa of abdominal organs, in particular on the intestine (Fig. 2). Although no obvious nodules were visible on the peritoneal surface, it appeared to be rough (Fig. 1).

The lungs were multifocally adhered to the costal pleura on both sides of the thorax. The left lung and half of the right lung were atelectatic.

Except from the fluid in the pericardial sac, no alterations were detected on the heart.

Further findings included rounded margins of the liver (Fig. 1) and several small colonic diverticula.

**Figure 3:**
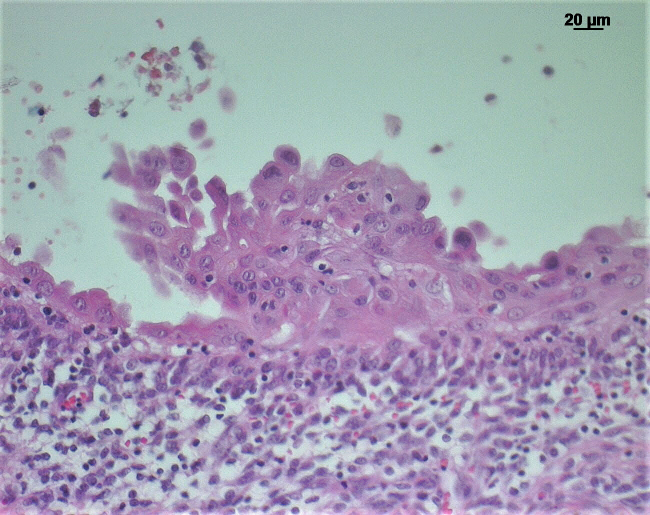
H&E-stained section of the jejunum of an African green monkey with reactive mesothelial hyperplasia: papillary projection of mesothelial cells on the intestinal serosa and subserosal inflammatory cell infiltration underneath.

### 3.2 Histopathology

On the serosal surfaces of the abdominal organs, the lungs, the pericardial sac, the heart, and the esophagus either multiple or single layers of plump mesothelial cells were detected. In part, mesothelial cells formed papillary projections (Fig. 3) but did not show tissue invasion. On the liver, the intestine, and especially in the thorax, the mesothelial cell layer was supported by a zone of connective tissue, infiltrated with lymphocytes, plasma cells, and – to a lesser degree – eosinophils (Fig. 3).

Mesothelial cells were characterized by an oval to polygonal shape with a large amount of eosinophilic cytoplasm, a single, large nucleus containing one nucleolus. Borders between the cells were distinct, and pyknosis and karyorrhexis of the nucleus were regularly observed. Single mitotic figures were visible within the mesothelial cell clusters.

Mesothelial cells were weakly positive with PAS reaction (mainly basal membrane and intercellular borders) and Alcian blue (mainly intercellular borders) and negative for Prussian blue.

No pathological gastrointestinal mucosal lesions were seen in this case.

### 3.3 Detection of asbestos

Fibers were not detectable within lung tissue.

**Figure 4:**
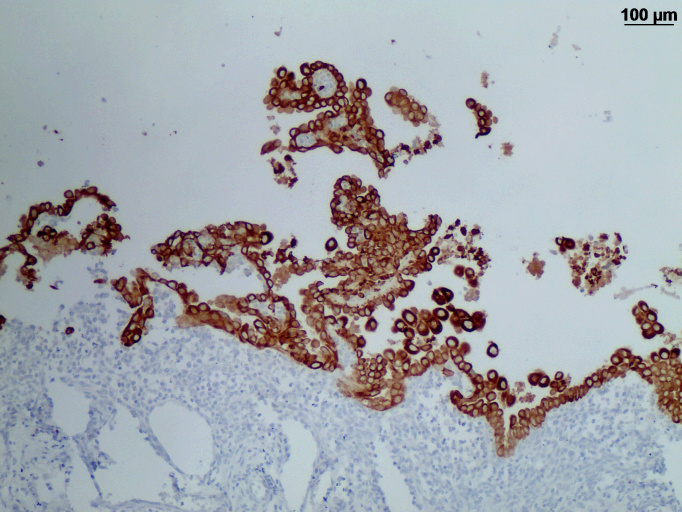
Immunohistochemistry of a reactive mesothelial hyperplasia in an African green monkey: strong immunoreactivity of the mesothelial cells for cytokeratin.

### 3.4 Immunohistochemistry

Mesothelial cells showed strong immunoreactivity for cytokeratin (Fig. 4) and were positive for vimentin, calretinin, desmin, and WT-1. Faint reactivity was seen with EMA immunohistochemistry. The cells were negative for CD15, CEA, and podoplanin.

In addition, the monkey was tested negative for SV40 immunohistochemically.

### 3.5 Transmission electron microscopy

**Figure 5:**
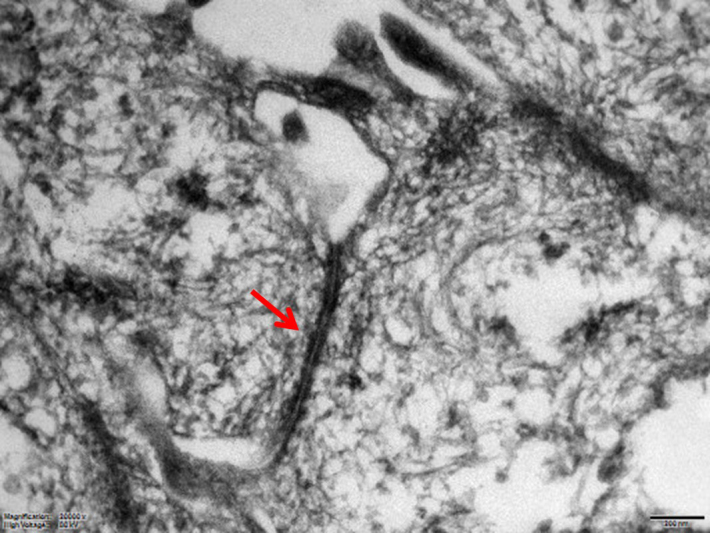
Electron microscopic image of a long desmosome (arrow) between mesothelial cells (scale bar: 200 nm).

Long desmosomes, perinuclear tonofilaments, and a small amount of microvilli of moderate length were demonstrated in mesothelial cells ultrastructurally (Fig. 5).

### 3.6 Genetic investigation

**Figure 6:**
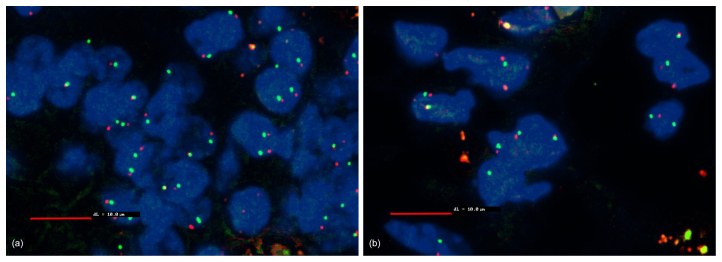
Fluorescence in situ hybridization (FISH) with MTAP (scale bar: 10 µm). Blue: DAPI staining of the cell nucleus. Red: Texas-red-stained MTAP probe. Green: FITC-stained CEN9q locus as control. Non-hyperplastic cells serve as a control. The CEN9q locus (green) localized close to the gene of interest (red) serves as a positive control to detect the chromosome pair. In a healthy cell, each nucleus should show two red and to green signals with a red and a green signal close together. If a deletion is present in a cell, only a single green signal of the CEN9q locus is visible. Some nucleoli might be cut between or even within chromosomes; therefore, a threshold of 10 % is defined for diagnosis of a deletion. No deletion detectable. **(a)** Non-hyperplastic cells of the jejunum; **(b)** mesothelial hyperplastic cells of the jejunum.

Using fluorescence in situ hybridization (FISH), no deletion of the 9p21 locus (p16; CDKN2A), NF2, and MTAP were observed (Fig. 6).

## 4 Discussion

Macroscopically, multiple small white nodules might be seen both in reactive mesothelial hyperplasia and in a mesothelioma (Watkins et al., 2018; Tischoff and Tannapfel, 2017). At necropsy, it is therefore impossible to distinguish both entities. In addition, effusions might occur in both types of alterations.

Histopathologically, a chronic polyserositis was demonstrated in this case. However, the identification of the underlying cause of a polyserositis might be a challenge. Polyserositis has been associated with different aetiologies, such as autoimmune, autoinflammatory, infectious, endocrine, metabolic, toxic origin, or neoplastic diseases (Losada et al., 2018). The occurrence of inflammatory cells like neutrophils in this case might give a hint for a bacterial infection. In principle, many different bacteria like Escherichia, Enterococcus, Streptococcus, Staphylococcus, Klebsiella, Corynebacterium, Clostridium, or Nocardia are able to contribute to/initiate a peritonitis. Their occurrence is often dependent on the point of entry (via intestine, via genital tract, or via traumatic location). Since the abdominal fluid was not further investigated and due to the chronicity of the inflammation in this case, it was impossible to identify the definite cause of the polyserositis. However, the occurrence of many eosinophilic granulocytes in this specific case might raise speculations about an autoimmune response or of parasites as a potential cause. Although they were not demonstrated in this particular case, in our monkey colony, amebae and balanthidiae are regularly identified. In general, pathogenic ameba species are known to have the potential to cause peritonitis (Toft and Eberhard, 1998).

Microscopically, it is often also a challenge to distinguish between benign mesothelial proliferation and malignant mesotheliomas. In single cases, it might even be impossible. Tissue invasion is a key criterion; however, it might be difficult to demonstrate since tissue invasion can occur in only very few areas and it may involve only few tumor cells. In our case, invasion of mesothelial cells into the underlying organs could not convincingly be demonstrated. This is consistent with human cases, in which it is often very difficult to find tissue infiltration.

Immunohistochemical staining patterns of mesotheliomas vary, and there is no single, reliable marker for mesotheliomas (Husain et al., 2018). In our case, the mesothelial cells were positive for cytokeratin/vimentin and negative for CEA. This indicates mesothelial origin and is confirmed by a positive reaction using calretinin and WT-1. This immunohistochemical staining profile is a helpful but not reliable tool to distinguish a mesothelial malignancy from a benign hyperplasia.

Fluorescence in situ hybridization (FISH) might help to demonstrate a homozygous p16 deletion, which is very characteristic for malignant mesotheliomas. One of the most common genetic alterations in humans is the homozygous deletion of the 9p21 locus (p16; CDKN2A). In up to 80 % of primary pleural malignant mesotheliomas and in up to 25 % of human peritoneal malignant mesotheliomas this deletion can be observed (Chiosea et al., 2008). Instead of CDKN2A fluorescence in situ hybridization, MTAP immunohistochemistry can be used in order to diagnose malignant mesotheliomas (Chapel et al., 2019). Somatic NF2 deletion also occur in a high proportion of human primary malignant mesotheliomas (Bianchi et al., 1995). However, in our case, these deletions could not be demonstrated, additionally indicating a reactive mesothelial hyperplasia instead of a mesothelioma. However, to our knowledge, this is the first case, in which a human FISH probe was used for the detection of genetic alterations in mesothelial proliferations in an African green monkey.

Ultrastructural features are also useful and reliable to diagnose malignant mesotheliomas (Husain et al., 2018). Human mesothelioma cells are characterized by very long thin apical microvilli, perinuclear tonofilament bundles, basal laminas, and long desmosomes (Oczypok and Oury, 2015; Husain et al., 2013; Hammar, 2006). In our case, we could only demonstrate a moderate amount of microvilli of medium length. However, perinuclear tonofilament bundles, and long desmosomes were present in our monkey mesothelial cells. The poor preservation status of the formalin-fixed and deparaffinized tissue probably impeded the detection of basement membranes in our case.

Another hint for the benign nature of this mesothelial proliferation is the fact that in humans, three main reasons for the development of mesotheliomas are known: contact to asbestos or other fibers, an infection with SV40, and radiation. However, each of these causes could be excluded in this case: (a) no asbestos fibers could be demonstrated in the lungs; (b) no SV40-antigen could be demonstrated in tissues of this monkey; and (c) there was no clinical history of radiation in this monkey.

## 5 Conclusion

This report describes a reactive mesothelial hyperplasia as a reaction to a polyserositis in an African green monkey (*Chlorocebus aethiops*) mimicking an epitheloid mesothelioma. Histopathology, immunohistochemistry, fluorescence in situ hybridization, and electron microscopy were used to determine the benign nature of the mesothelial proliferation. For the first time, human genetic probes had been successfully applied to an African green monkey.

## Data Availability

Remaining paraffin-embeded organ material is available via the corresponding author.
